# Polyphosphate-crosslinked collagen scaffolds for hemostasis and alveolar bone regeneration after tooth extraction

**DOI:** 10.1016/j.bioactmat.2021.12.019

**Published:** 2021-12-26

**Authors:** Jun-ting Gu, Kai Jiao, Jing Li, Jian-fei Yan, Kai-yan Wang, Fu Wang, Yan Liu, Franklin R. Tay, Ji-hua Chen, Li-na Niu

**Affiliations:** a National Clinical Research Center for Oral Diseases, State Key Laboratory of Military Stomatology, Shaanxi Key Laboratory of Stomatology, Department of Prosthodontics, School of Stomatology, The Fourth Military Medical University, Xi'an, Shaanxi, China; b Department of Endodontics, The Dental College of Georgia, Augusta University, Augusta, GA, 30912, USA

**Keywords:** Alveolar ridge preservation, Blood clotting, Osteogenesis, Polyphosphate

## Abstract

Post-extraction bleeding and alveolar bone resorption are the two frequently encountered complications after tooth extraction that result in poor healing and rehabilitation difficulties. The present study covalently bonded polyphosphate onto a collagen scaffold (P-CS) by crosslinking. The P-CS demonstrated improved hemostatic property in a healthy rat model and an anticoagulant-treated rat model. This improvement is attributed to the increase in hydrophilicity, increased thrombin generation, platelet activation and stimulation of the intrinsic coagulation pathway. In addition, the P-CS promoted the *in-situ* bone regeneration and alveolar ridge preservation in a rat alveolar bone defect model. The promotion is attributed to enhanced osteogenic differentiation of bone marrow stromal cells. Osteogenesis was improved by both polyphosphate and blood clots. Taken together, P-CS possesses favorable hemostasis and alveolar ridge preservation capability. It may be used as an effective treatment option for post-extraction bleeding and alveolar bone loss.

**Statement of significance:**

Collagen scaffold is commonly used for the treatment of post-extraction bleeding and alveolar bone loss after tooth extraction. However, its application is hampered by insufficient hemostatic and osteoinductive property. Crosslinking polyphosphate with collagen produces a modified collagen scaffold that possesses improved hemostatic performance and augmented bone regeneration potential.

## Highlights

• Polyphosphate-crosslinked collagen scaffold (P-CS) showed better hemostatic effect in healthy or anticoagulant-treated rats.
• The promoted bone regeneration ability of P-CS might also be related to the clot alteration caused by polyphosphate.
• P-CS has therapeutic potential in bleeding control and alveolar ridge preservation after tooth extraction.

## Graphical abstract

**Figure:**
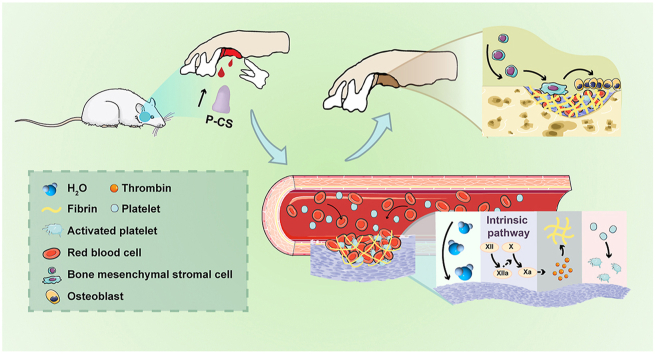

## 1 Introduction

Post-extraction bleeding and alveolar bone resorption are two frequently encountered complications after tooth extraction. Post-extraction bleeding is defined as bleeding continuously beyond 8–12 h after extraction [1]. Its incidence varies from 0% to 26%, and is especially high in those individuals taking anticoagulants [[1], [2], [3]]. Without suitable management, post-extraction bleeding may result in severe complications. Different hemostatic agents are used as local intervention for treating post-extraction bleeding [1]. However, existing hemostatic agents are incapable of meeting the demand for hemostasis in clinical practice, especially for patients undergoing long-term anticoagulant therapy such as warfarin for the prevention of strokes or recurrent venous thromboembolic events [4].

Alveolar bone resorption often results in compromised prosthodontic rehabilitation outcomes. A mean reduction of 3.8 mm in alveolar bone width and 1.24 mm in alveolar bone height has been reported after tooth extraction [5]. To solve this problem, alveolar ridge preservation is introduced to decrease resorption and facilitate the post-extraction prosthodontic and aesthetic restoration [6,7]. Alveolar ridge preservation involves the application of bone graft materials, with or without a barrier membrane, to the extraction sockets. This technique aims at minimizing external resorption of the alveolar ridge and maximizing bone formation within the socket. Bone graft materials consist of autografts, allografts, xenografts, as well as synthetic biomaterials [6]. Both allogeneic and xenogeneic bone transplantation may generate varying degrees of antigen reaction. Hence, the majority of the research conducted on bone graft materials have been focused on biomaterial scaffolds [[8], [9], [10], [11]].

Because post-extraction bleeding and alveolar bone resorption often lead to poor healing outcomes and compromises in prosthodontic rehabilitation, solutions that capable of solving both complications are urgently needed. Collagen is the most abundant extracellular matrix protein and is highly biocompatible. It has been used extensively for different biomedical applications [12]. Collagen possesses hemostatic and osteoinductive properties [13,14]. It is an excellent biomaterial for bleeding control and alveolar ridge preservation after tooth extraction [7]. One of the limitations of collagen is its poor enzyme resistance, which may be solved by crosslinking [15]. Chemical crosslinking can bind functional groups onto collagen fibrils and endow a collagen scaffold with desirable biological properties [16]. This treatment may be used for improving the hemostatic, bone regenerative and enzyme resistant properties of collagen scaffolds.

Inorganic polyphosphate is an easily obtainable polymer that consists of linear chains of orthophosphate residues [17]. It promotes blood clotting by accelerating the activation of factor XI and increasing the generation of thrombin [18]. Polyphosphate also induces the proliferation and osteogenic differentiation of mesenchymal stem cells. It is used extensively in bone regeneration to expedite bone repair [19]. Hence, polyphosphate is a rational choice for collagen modification. Accordingly, the objective of the present study was to test the hypothesis that polyphosphate can be crosslinked onto collagen for enhancement of bleeding control and bone repair.

## 2 Materials and methods

All chemicals were purchased from MilliporeSigma (Burlington, MA, USA) except when specified. Blood was drawn from the abdominal aorta of Sprague-Dawley rats and mixed with an anticoagulant (acid citrate dextrose) at a 9:1 v/v ratio. The rats were obtained from the Laboratory Animal Research Center of the Fourth Military Medical University, Xi'an, China and kept under pathogen-free conditions. The animal protocols were approved by the Institutional Animal Care and Use Committee of the Fourth Military Medical University (IACUC-20200314) and met the National Institute of Health guidelines for the care and use of laboratory animals. Group designation and experimental scheme are described in Fig. S1 (Supplementary material).

### 2.1 Polyphosphate-crosslinked collagen scaffolds (P-CS)

#### 2.1.1 Preparation

Ammonium polyphosphate (MW ∼22 kDa, Macklin, Shanghai, China) is a common inorganic polyphosphate. It was covalently bonded to collagen scaffolds (Ace Surgical Supply Co., Inc, MA, USA) via 1-ethy-3(3-dimethylaminpropyl)-carbodiimide (EDC)/N-hydroxysulfosuccinimide (NHS) crosslinking. Based on the results from a pilot study, 200 mg of ammonium polyphosphate was dissolved in 50 mL of 0.1 M 2-(N-morpholino) ethane-sulfonic acid buffer (pH 6.0) and mixed with 20 mg of EDC and 55 mg of NHS for 30 min. The pH of the mixture was increased to 7.3 using concentrated phosphate-buffered saline (PBS; pH 7.2–7.5). Collagen scaffolds were incubated with the solution for 30 min at room temperature and then washed with Milli-Q water, lyophilized and used as polyphosphate-crosslinked collagen scaffolds (P-CSs). All specimens were disinfected using cobalt-60 irradiation prior to further investigation [20].

#### 2.1.2 Crystal violet staining

Because of the presence of anionic phosphate groups on polyphohsphate, a cationic dye was used to test the binding of polyphosphate to collagen scaffolds. Crystal violet staining was conducted as previously described [20]. Briefly, the pristine, non-crosslinked scaffolds and P-CSs were stained with 0.1% (w/v) crystal violet for 10 min and thoroughly rinsed with a destainer (acetic acid: ethyl alcohol: H_2_O = 2:1:17 v/v) for 10 min (n = 6).

#### 2.1.3 Ruthenium red staining

A single layer of collagen fibrils was reconstituted on Ni grids (Grade V1, Ted Pella Inc., CA, USA), in the manner reported by Song et al. [16]. The pristine collagen fibrils were crosslinked with ammonium polyphosphate in the manner described in 2.1.1. Nickel grids coated with single layers of pristine collagen or polyphosphate-crosslinked collagen (n = 6) were stained with 0.02% (w/v) ruthenium red. The latter was dissolved in 0.1 M sodium cacodylate buffer (pH 7.2). The stained grid was examined with a JEM-1230 transmission electron microscope (TEM; JEOL, Tokyo, Japan) at 110 kV [16].

#### 2.1.4 Scanning electron microscopy

Pristine collagen scaffolds and P-CSs were dehydrated with an ascending ethanol series (15 min each in 50%, 70%, 80%, 90% and 100% ethanol), incubated for 30 min in hexamethyldisilazane (HMDS) and slowly air-dried. The specimens (n = 6) were sputter-coated with gold/palladium and examined with a field-emission scanning electron microscope (FE-SEM, S-4800, Hitachi, Tokyo, Japan) at 5 kV. Elemental analysis was performed using energy dispersive X-ray spectroscopy (EDAX).

#### 2.1.5 Attenuated total reflection-Fourier transform infrared spectroscopy

Infrared spectra of pristine scaffolds and P-CSs were collected using attenuated total reflection-Fourier transform infrared spectroscopy (ATR-FTIR; FTIR-8400S, Shimadzu, Tokyo, Japan) with a resolution of 4 cm^−1^ from 400 to 4000 cm^−1^. The spectrum of P-CS was normalized against that of the collagen scaffold along the amide I peak (∼1640 cm^−1^).

#### 2.1.6 X-ray photoelectron spectroscopy

The surface elements of the pristine scaffolds and P-CSs were analyzed with X-ray photoelectron spectroscopy (XPS; K-Alpha, Thermo Fisher Scientific, Waltham, MA, USA), and the XPS spectra of P 2p region in P-CS was further analyzed.

### 2.2 Physical properties of P-CS

#### 2.2.1 Water contact angle

The water contact angles of the pristine scaffolds and P-CSs (10 mm diameter, 2 mm thick) were evaluated with a contact angle goniometer (Easy Drop K100, Kruss Co., Hamburg, Germany). Water droplets were pumped out of the syringe at a rate of 2.00 μL/s. Water sorption by the specimen was recorded. At the moment a water droplet contacted the specimen, the contact angle was measured and averaged (n = 6). The duration for a specimen to absorb one droplet of water was recorded (n = 6).

#### 2.2.2 Water sorption

The pristine scaffolds and P-CSs were pre-weighed prior to immersion in Milli-Q water for 5 s. The wet specimens were weighed after the water stopped dripping upon removal (n = 6). Water sorption was calculated as:

Water absorption ratio = (W_wet_ − W_dry_)/W_dry_

where W_dry_ is the weight of dry specimen and W_wet_ is the weight of wet specimen [13].

#### 2.2.3 Expansion ratio

The pristine scaffolds and P-CSs were cut into cubes, the dimensions of which were measured with a digital micrometer (Mitutoyo, Kanagawa, Japan). The specimens were immersed into water for 5 s. Excess water on the surface of collagen was removed. The dimension of the wet specimen was measured (n = 6). Expansion ratio was calculated as:

Expansion ratio = (V_wet_ − V_dry_)/V_dry_

where, V_dry_ is the volume of dry sample and V_wet_ is the volume of wet sample.

#### 2.2.4 Modulus of elasticity

The pristine scaffolds and P-CSs were cut into 40 × 5 × 2 mm^3^ (length × breadth × height) blocks. Each specimen was fixed between the two opposing grips of a tensile tester (Shimadzu). Each specimen was stressed to failure under tension, using a crosshead speed of 1 mm/min (n = 6). The Young's modulus was calculated as the slope of the linear region in the respective stress-strain curve.

#### 2.2.5 Hydroxyproline assay

Pristine collagen scaffolds and P-CSs with the same initial weight were incubated in 0.1 mg/mL type I collagenase (MilliporeSigma) at 37 °C. After 0, 5, 10, 15, 20 or 25 min, the remnant materials were collected. A hydroxyproline assay kit (MilliporeSigma) was used to quantify the collagen content in the remnant materials (n = 6). Degradation ratio was obtained using the equation:

Degradation ratio = (C_0_ − C_t_)/C_0_*100%

where C_0_ is the collagen content of samples before enzymatic hydrolysis and C_t_ is the collagen content in specimens that were enzymatically hydrolyzed for a designated time-period.

### 2.3 Biocompatibility of P-CS

#### 2.3.1 Hemolysis

The hemolytic property of the pristine scaffolds or P-CSs was evaluated as described elsewhere [21]. Briefly, the scaffolds (4 mm diameter and 2 mm thick) were pre-warmed at 37 °C for 30 min prior to the experiment. One hundred microliter of blood containing the anticoagulant was added to the scaffolds. After 30 min, 1 mL of normal saline solution was added to stop the hemolysis. The mixtures were incubated at 37 °C for 30 min and centrifuged at 600 g for 15 min. Optical density values of the supernatants were detected at 540 nm using a microplate reader (BIO-TEK, Winooski, VT, USA). Blood containing no scaffold served as negative control. Blood treated with 1 mL of Triton X-100 (MilliporeSigma), a non-ionic surfactant, served as positive control.

#### 2.3.2 Cytotoxicity

Human oral fibroblasts (ATCC, Manassas, VA, USA, 15 passage) were cultured with collage scaffolds or P-CSs for 24, 48 or 72 h. A cell counting kit-8 (CCK-8, Dojindo Molecular Technologies, Rockville, MD, USA) was used to evaluate cell viability at the designated time-period (n = 6). The plate was read at 450 nm using the microplate reader. Cells without exposure to scaffolds were used as positive control. Cell medium without cells was used as negative control. Cell viability was determined using the formula:

Cell viability = (OD_test_ − OD_negative_)/(OD_positive_ − OD_negative_)

where, OD_test_ is the optical density of the test sample, OD_negative_ and OD_positive_ stand for the OD value of the negative control and the positive control, respectively.

### 2.4 Hemostasis *in vitro*

#### 2.4.1 Dynamic whole blood clotting assay

Pristine collagen scaffolds or P-CSs (4 mm diameter and 2 mm thick) were pre-warmed at 37 °C for 30 min prior to experiment. One hundred microliter of anticoagulant-containing whole blood was added to each type of scaffolds. Another 10 μL of 0.2 M CaCl_2_ was added immediately. One microliter of Milli-Q water was added to the specimens at 5, 15 or 30 min to hemolyze the unclotted red blood cells. After incubation at 37 °C for 15 min, the liquid from each specimen was centrifuged at 180 g for 1 min. Optical density values of the supernatants were detected at 540 nm. Blood cultured with no scaffolds served as negative control. The blood clotting ratio was calculated as:

Blood clotting ratio = (OD_negative_ − OD_test_)/OD_negative_*100%

where OD_test_ is the absorbance of supernatant in the test group and OD_negative_ is the absorbance of the supernatant in the negative control.

#### 2.4.2 SEM of blood clots

Both scaffolds were incubated individually in whole blood at 37 °C for 30 min. The scaffolds were washed with PBS and fixed in 2.5% glutaraldehyde for 2.5 h prior to processing for SEM examination (n = 6) [22].

#### 2.4.3 Platelet adhesion

Platelet-rich plasma was prepared by centrifuging anticoagulant-containing blood at 800 rpm for 10 min. The scaffolds were incubated in platelet-rich plasma at 37 °C for 30 min prior to processing for SEM examination [23].

#### 2.4.4 Platelet activation

Platelet activation was evaluated using P-selectin expression. Whole blood was incubated with the scaffolds at 37 °C for 30 min (n = 6). Soluble P-selectin in the plasma was quantified by enzyme-linked immunosorbent assay (ELISA; Rat P-Selectin ELISA kit, Cusabio, Wuhan, P.R. China) [24].

#### 2.4.5 Thrombin generation

Platelet-poor plasma was prepared by centrifuging anticoagulant-containing blood at 3000 rpm for 15 min. The platelet-poor plasma was incubated with the scaffolds at 37 °C for 5, 15 or 30 min (n = 6). Two hundred microliter of platelet-poor plasma was removed and incubated with 200 μL of chromogenic substrate S2238 (5 Mm, Aglyco, Beijing, China) at 37 °C for 5 min. The OD value was detected at 405 nm. Thrombin generation was calculated from a standard curve of thrombin concentration *vs* OD values.

#### 2.4.6 Coagulation time

Platelet-poor plasma was incubated with the specimens at 37 °C for 3 min. The prothrombin time (PT) and activated partial thromboplastin time (APTT) were detected with a semi-automatic coagulation analyzer (RT-2202; Rayto Life and Analytical Science, Shenzhen, China) with a PT test kit and an APTT test kit (Yaji Biotechnology Co., Ltd., Shanghai, China).

### 2.5 Hemostasis *in vivo*

#### 2.5.1 Warfarin-treated rat model

The oral anticoagulant warfarin was used to develop a rat coagulation disorder model. Briefly, 6-week-old Sprague-Dawley rats were given warfarin (Warfarin sodium; Xinyi Pharmaceutical Co, Shanghai, China) 0.2 mg/kg per day via gavage for 7 days [25]. Rats that received normal saline solution was used as healthy control. Each group consists of 18 rats and were used in the studies described below. Anesthesia was performed by intraperitoneal injection of sodium pentobarbital (30 mg/kg).

In the following three models, a double-blinded design was applied to minimize the bias. The surgeon performing the surgery was unaware of the assigned intervention (first blind). The intervention (P-CS or pristine collagen scaffold) was administered by another surgeon. Evaluation of blood loss and bleeding time were performed by individuals who were unaware of the intervention (second blind). Because P-CS and pristine collagen scaffold cannot be distinguished in the appearance, the blinded status of experimental evaluators was ensured.

#### 2.5.2 Hepatic trauma model

The hepatic trauma model was used to evaluate the hemostasis effect of P-CS on parenchymatous hemorrhage. After anesthesia, each rat was fixed with its abdomen facing upward. The anterior hepatic lobe was adequately exposed using laparotomy. A 2 cm-long incision was created using a scalpel to initiate bleeding. A collagen scaffold (control) or P-CS was then placed over the cut (n = 6). The time required for hemostasis was recorded when bleeding started to stop. The spilled blood was blotted by a piece of gauze. Blood loss was calculated by measuring the weight changes of the gauze and scaffolds [22].

#### 2.5.3 Femoral artery injury model

The femoral artery injury model was used to evaluate the hemostasis performance of P-CS on arterial hemorrhage. After anesthesia, a unilateral femoral artery of each rat was exposed and punctured with a 20-gauge needle. The control collagen scaffold or P-CS was placed on the crevasse (n = 6). The time required for hemostasis and the blood loss were obtained using the same method described in 2.5.2. [13].

#### 2.5.4 Tooth extraction model

The tooth extraction model was used to evaluate the hemostasis performance of P-CS on post-extraction bleeding. After anesthesia, the gingiva around the right maxillary first molar was carefully separated with a dental probe. After extraction of the tooth, the socket was filled with the collagen scaffold control or the P-CS to stop bleeding (n = 6). The time required for hemostasis and the blood loss were obtained using the same method described in 2.5.2.

### 2.6 Bone regeneration *in vivo*

#### 2.6.1 Rat alveolar bone defect model

The rat alveolar bone defect model was used to evaluate the bone regeneration potential of P-CSs [26,27]. Briefly, the right maxillary first molar was extracted as described in section 2.5.4. A standardized defect was created immediately after tooth extraction, using a 1.4 mm-diameter dental bur with normal saline irrigation. Evacuation was performed to a depth of 2 mm from the cementoenamel junction of the right maxillary second molar. Pristine collagen scaffolds or P-CSs were implanted into the extraction socket. Sockets without grafts served as negative control. The surrounding mucosal tissue was sutured following scaffold placement to achieve primary closure. The surgical procedure was shown in Fig. S7

#### 2.6.2 Micro-computed tomography (Micro-CT)

The rats were sacrificed at 7^th^ and 21^st^ day after surgery (n = 6), by intraperitoneal injection of pentobarbital sodium (40 mg/kg). The maxilla was dissected and fixed in 4% paraformaldehyde. The specimens were scanned using micro-CT (Inveon, Siemens Preclinical, Knoxville, TN, USA) at high resolution. Images were reconstructed into a three-dimensional structure and analyzed using the Inveon Research Workplace software. Sagittal slices were used to evaluate new bone formation in the maxilla. The region of interest was defined as the first slice in which the defect was observed to the last slice in which the defect was no longer observed. New bone volume fraction (bone volume/total volume, BV/TV) and bone mineral density (BMD) in the defect site at 7^th^ and 21^st^ day, as well as changes in alveolar bone height and ridge width at the 21^st^ day were calculated as previously described. Bone height was measured by recording the distance from the upper-most of the maxillary alveolar process to the bottom-most of the buccal or palatal alveolar crest. Ridge width was measured by the greatest bucco-palatal width at 1.25 mm above the most lateral point of the buccal cortical plate [8,26].

#### 2.6.3 Histology and histomorphometry

The maxillary bones from rats scarified at 21^st^ day after surgery were fixed with 4% paraformaldehyde (n = 6). After dehydration using an ascending series of ethanol, the specimens were infiltrated with purified methyl methacrylate and polymerized. The non-decalcified specimens were sectioned longitudinally at a thickness of ∼50 μm (Leica SP1600, Mannheim, Germany). The sections were stained with Goldner's trichrome, von Kossa and Masson's trichrome (all from Servicebio, Wuhan, Hubei, China) to analyze morphological changes in the defect sites. For Goldner's trichrome, green areas (indicative of mineralized bone) in the region of interest were considered positive. For von Kossa staining, dark brown areas (indicative of calcified nodules) were considered positive. For Masson trichrome, blue areas (indicative of collagen fibrils) were considered positive. The positive area fractions (positive areas/total areas; PA/TA) were measured using ImageJ software (National Institute of Health, Bethesda, MD, USA) and analyzed [20,28,29].

### 2.7 Interaction between blood clots and bone marrow mesenchymal stem cells (BMSCs)

The BMSCs were harvested from 4-week-old Sprague-Dawley rats, authenticated by CD44 (15675-1-AP; Proteintech, Rosemont, IL, USA) and expanded as previously reported [30]. Cells from passage 4–6 were used for study. The culture medium was α-MEM (Gibco, ThermoFisher Scientific) containing 10% fetal bovine serum and 1% penicillin/streptomycin (Invitrogen, ThermoFisher Scientific). The collagen sponges or P-CSs were pre-incubated with whole blood at 37 °C for 30 min prior to further use.

#### 2.7.1 Transwell migration assay

The effects of blood clots on cell migration were examined using the transwell migration assay (Millicell Standing Cell Culture Inserts, 8-μm pore size; Merck-Millipore, Burlington, MA, USA). The BMSCs were seeded in the upper chamber of each Transwell at a density of 8 × 10^4^ cells/well (Fig. 7A). The inserts were then placed into 24-well plates that were filled with α-MEM and the designated specimens (collagen sponge, P-CS, collagen sponge+blood, P-CS+blood; n = 6). After 24 h, the content of P-selectin in the lower chamber was analyzed by ELISA. The upper chamber was removed, fixed with 4% paraformaldehyde and stained with 0.2% crystal violet. Cells adhering to the membrane inside the inserts were gently removed using a cotton-tipped applicator. Migrated cells on the other side of the inserts were imaged using light microscopy (DM4000 B; Leica Microsystems, Wetzlar, Germany) and counted with the ImageJ software.

#### 2.7.2 Immunofluorescence staining

The BMSCs were seeded on both bare or blood pre-incubated collagen sponges and P-CS for 12 h (n = 6), as shown in Fig. 7D. The cells were fixed with 4% paraformaldehyde and permeabilized for in 0.15% Triton X-100. The cytoskeleton was stained with Alexa Fluor 488-labeled phalloidin (1:40, Invitrogen, Switzerland). All specimens were rinsed and mounted with Prolong Diamond Antifade Mountant with 4’,6-diamidino-2-phenylindole (DAPI; Invitrogen, ThermoFisher Scientific). The images were captured using confocal laser-scanning microscopy (Nikon A1R, Nikon Corporation, Minato-ku, Tokyo, Japan). Cell spreading area was calculated using Image J software.

#### 2.7.3 SEM

The interaction between BMSCs and clots was further investigated by SEM. Cell incubation was conducted as described in 2.7.2 and examined with SEM.

#### 2.7.4 Reverse transcription-polymerase chain reaction (RT-PCR)

This procedure was used to investigate the osteogenic differentiation of BMSCs. When the BMSCs reached 80% confluency, the α-MEM culture medium was replaced by osteogenic induction medium. The latter consisted of 1 nM dexamethasone, 50 μM l-ascorbic acid-2-phosphate and 20 mM β-glycerophosphate. The BMSCs were seeded on pristine scaffolds or P-CS, with or without blood pre-incubation. The cells were cultured in osteogenic induction medium for 14 days. Cells seeded on pristine scaffolds were used as control (n = 6).

Three osteogenesis-related genes were examined: *runt-related transcription factor 2* (*RUNX-2*)*, osterix* and *osteocalcin (OCN)*. The primer sequences used for RT-PCR are shown in Supplementary Table 1. Total RNA was isolated using Trizol reagent and reverse-transcripted using the Prime Script RT reagent kit (Takara Bio Inc., Shiga, Japan). Real-time PCR (7500 Real-time PCR System; Applied Biosystems, Carlsbard, CA, USA) was performed with sense and antisense primers based on published cDNA sequences. Glyceraldehyde 3-phosphate dehydrogenase was used as the housekeeping gene. Fold changes relative to the control group were estimated using the 2^-△△CT^ method [20].

### 2.8 Statistical analyses

All data were presented as means ± standard deviations. The Shapiro-Wilk test was used to test for the normality assumption of the corresponding data sets. The modified Levene test was used to test the homogeneity of variance assumption of the corresponding data sets. Parametric statistical tests were used after validating that those two assumptions were not violated. Comparisons between 2 groups were examined using Student's t-test. Comparisons involving more than 2 groups were analyzed using one-way analysis of variance (ANOVA) and post-hoc Tukey test for pairwise comparisons. The interaction between blood clots and BMSCs was analyzed using two-way ANOVA. The GraphPad Prism 5 package (GraphPad Software, La Jolla, CA, USA) was used for analyses. For all analyses, statistical significance was pre-set at α = 0.05.

## 3 Results

### 3.1 Characterization of P-CS

**Fig. 1:**
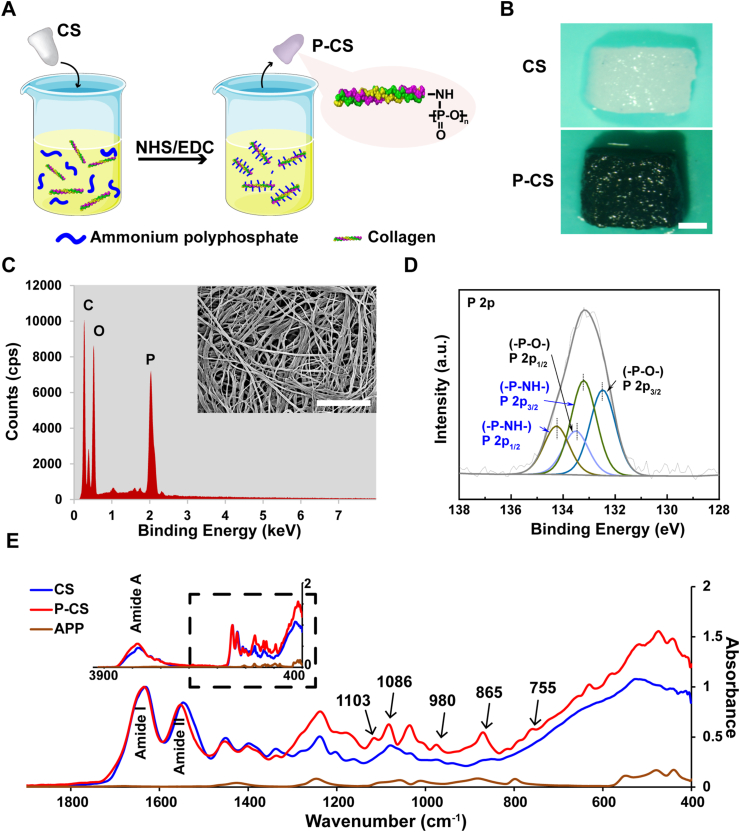
Characterization of polyphosphate-crosslinked collagen scaffold (P-CS). **A**. Schematic of the synthesis of P-CS. **B**. Crystal violet staining of pristine collagen scaffold (CS) and P-CS. Bar: 5 mm. **C**. SEM-EDAX spectrum of P-CS. Bar: 5 μm. **D**. XPS spectrum of P 2p region of P-CS. **E**. ATR-FTIR spectra of ammonium polyphosphate (APP), CS and P-CS. The parts of the spectra outlined in the square inset were magnified for clarity. The spectra of CS and P-CS was normalized along the collagen amide I peak (∼1640 cm^−1^). Peaks associated with amide A (∼3300 cm^−1^), amide I and amide II (∼1545 cm^−1^) are characteristic of collagen.

Ammonium polyphosphate is an inorganic polyphosphate rich in phosphate radicals. Covalent binding of ammonium polyphosphate to collagen was conducted using EDC/NHS crosslinking (Fig. 1A). The anionic phosphate is stainable by cationic dyes. Crystal violet staining showed that the P-CS was stained purple whereas a pristine collagen sponge was unstained (Fig. 1B). Ruthenium red staining showed that staining was miniscule on the surface of pristine collagen fibrils, whereas staining was profuse on the surface of polyphosphate-crosslinked collagen fibrils (Fig. S2). Staining with these two cationic dyes confirmed that polyphosphate has been successfully conjugated on the surface of the P-CSs. The EDAX spectrum of P-CS showed a high peak of P (Fig. 1C). This indicates that abundant P elements are present on the P-CS surface.

Fig. 1E shows the infrared spectra of a collagen sponge and a P-CS. In these spectra, type I collagen is represented by the characteristic amide A (∼3300 cm^−1^), amide I (∼1640 cm^−1^) and amide II (∼1545 cm^−1^) peaks. Observation of these peaks in the P-CS specimen indicates that the collagen structure was not damaged after crosslinking with polyphosphate. New peaks appeared in the P-CS spectrum. The characteristic signals of polyphosphate were seen as the symmetric stretching vibration (ν_sym_) of P–O–P at ∼755 cm^−1^, the asymmetric stretching vibration (ν_as_) of P–O–P at ∼865 cm^−1^ and the ν_as_ vibration of (PO_3_)^2−^ at ∼1103 cm^−1^ [17]. The peaks at ∼980 cm^−1^ and ∼1086 cm^−1^ were assigned to the phosphoramide bond [31] and P–N–C [32], respectively.

In the XPS spectra, a peak that occurred in the P 2p region of the P-CS spectrum was not apparent in the spectrum of pristine collagen scaffold (Fig. S3). The P 2p region of P-CS exhibited functional groups -P-O- and -P-N- (Fig. 1D). These signals are indicative of the formation of stable chemical bonds between polyphosphate and collagen. Thus, the mechanism of polyphosphate and collagen binding is possibly attributed to the anchoring of polyphosphate molecule on the amino group of collagen through the formation of a phosphoramide bond.

### 3.2 Physical properties of P-CS

**Fig. 2:**
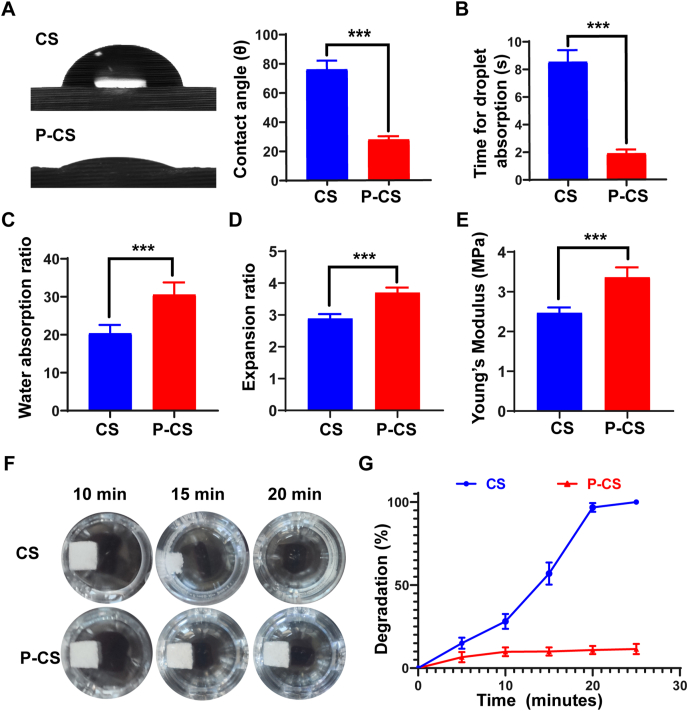
Physical properties of P-CS. **A**. Images and quantitative analysis of water contact angle on pristine collagen scaffolds (CS) and polyphosphate-crosslinked collagen scaffolds (P-CS) at the time a water droplet contacted the specimens. **B**. Time for CS and P-CS to absorb a water droplet. **C**. Water absorption ratio, **D**. volume expansion ratio, **E**. Moduli of elasticity (Young's moduli) of the different scaffolds. **F**. Observation and **G**. estimation of the percentage degradation of CS and P-CS after enzymatic hydrolysis. Data represent means and standard deviations; ***: p < 0.001 (Student's t-test; n = 6).

Contact angle measurement was used to analyze the difference in hydrophilicity of pristine collagen scaffolds and P-CSs. As shown in Fig. 2A, the contact angle of P-CS was significantly decreased as a water droplet contacted the material. The time for P-CS to absorb a droplet was also shorter than that of the control collagen scaffold (Fig. 2B). The P-CS exhibited higher water sorption ratio (Fig. 2C) and volume expansion ratio (Fig. 2D) after contacting with water for a short time. All the above results indicate increase in hydrophilic and hygroscopic characteristics of the P-CS. Hemostatic scaffolds should have sufficient mechanical strength to form stable hemostatic plugs. As indicated in Fig. 2E, the P-CS had a higher modulus of elasticity than the pristine collagen scaffold.

The degradation ratio of pristine scaffolds and P-CS was recorded at predetermined time-points. After enzymatic hydrolysis by collagenase, the pristine collagen scaffolds were almost completely degraded after 20 min (Fig. 2F and G). In contrast, the P-CSs had a low degradation rate of ∼10%. These results indicate that the P-CSs had improved resistance to enzymatic hydrolysis.

### 3.3 Biocompatibility of P-CS

Blood is the first component to contact an implanted collagen scaffold. Scaffolds with poor biocompatibility will cause lysis of red blood cells. Hence, the *in vitro* hemolysis assay was performed to evaluation the hemolytic activity of pristine collagen scaffolds and P-CS. As shown in Fig. S4A, both the control and crosslinked scaffolds showed no statistical difference with the normal saline negative control. With respect to the CCK-8 assay, there was no difference in cell viability between the two types of collagen scaffolds at 24 h, 48 h and 72 h (Fig. S4B).

### 3.4 Hemostasis of P-CS *in vitro*

**Fig. 3:**
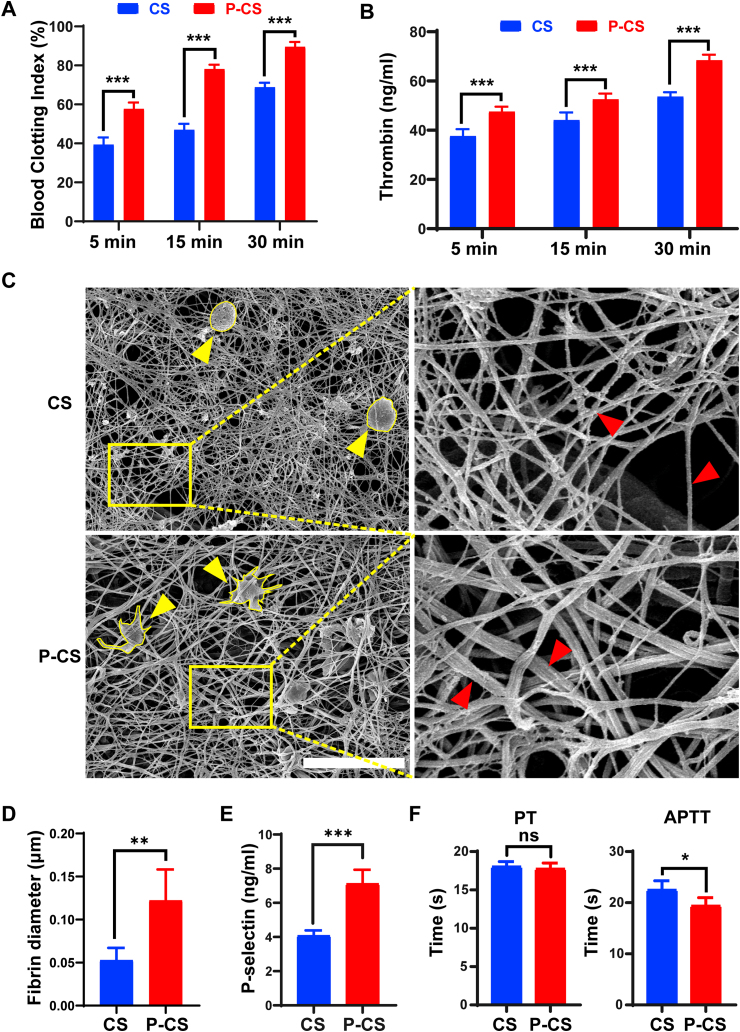
Hemostasis of P-CS *in vitro*. **A**. Dynamic blood clotting index of pristine collagen scaffolds (CS) and polyphosphate-crosslinked collagen scaffolds (P-CS) after clotting for 5, 15 or 30 min. **B**. Thrombin generation of CS and P-CS at different time points. **C**. Representative SEM images of platelets and fibrin on different specimens. Platelet morphology was outlined in yellow (yellow arrows). Images in yellow rectangles were magnified to show the difference in fibrin formation on CS or P-CS. Red arrows: fibrin strands. Bar: 5 μm. **D**. The diameter of fibrin formed on CS and P-CS. **E**. P-selectin secretion by whole blood after treated with different scaffolds. **F**. Influence of CS and P-CS on the prothrombin time (PT) and activated partial thromboplastin time (APTT). Data represent means and standard deviations; ns: no significant difference; *: p < 0.05; **: p < 0.01; ***: p < 0.001 (Student's t-test, n = 6).

The hemostatic property of P-CS was evaluated using the blood clotting assay. As shown in Fig. 3A, the blood clotting ratio of P-CS was more than that of pristine collagen scaffolds at all time-periods. After clotting was initiated for 30 min, the blood clotting index in the P-CS group was around 90%, which was close to complete coagulation. The APTT and PT tests are used for screening the activation of intrinsic and extrinsic pathway of coagulation, respectively. As shown in Fig. 3F, the APTT of P-CS was shortened, while the PT values did not show significant difference between the pristine scaffolds and P-CS. These results imply that the hemostatic property of P-CS was enhanced by the intrinsic coagulation pathway.

Scanning electron microscopy of whole blood that was incubated with pristine scaffolds or P-CS *in vitro* showed well-formed blood clots and deformed platelets on the P-CS (Fig. S5). Fibrin networks were produced to reinforce the thrombus and halt bleeding. Thrombin is a key factor in hemostasis that converts fibrinogen into fibrin. Hence, the ability of P-CS to generate thrombin was further investigated. After clotting for 5, 15 and 30 min, the thrombin generated in the P-CS group was more than that in the pristine collagen scaffolds (Fig. 3B).

When the pristine scaffolds and P-CSs were incubated with platelet-poor plasma, there was thicker fibrin formation on the P-CSs (Fig. 3D). A lot of deformed platelets were found on the surface of P-CS (Fig. 3C). Those platelets had their parapodia stretched out, which is indicative of platelet activation. Platelet activation was also evaluated by quantitatively measurement of the secretion of P-selectin, a marker for platelet activation. Secretion of P-selectin by platelets was increased in the P-CS group (Fig. 3E). The results demonstrate that P-CSs enhance platelet activate which, in turn, results in more rapid blood clotting.

### 3.5 Hemostasis of P-CS *in vivo*

Because post-extraction bleeding often occurs in the patients taking anticoagulants, a coagulation disordered model was established by treating rats with the anticoagulant warfarin. The warfarin-treated rats showed impeded coagulation, with significantly prolonged APTT and slightly prolonged PT (Fig. S6). Hemostasis of P-CS *in vivo* was evaluated using three different models in both normal saline-treated control rats and warfarin-treated rats.

**Fig. 4:**
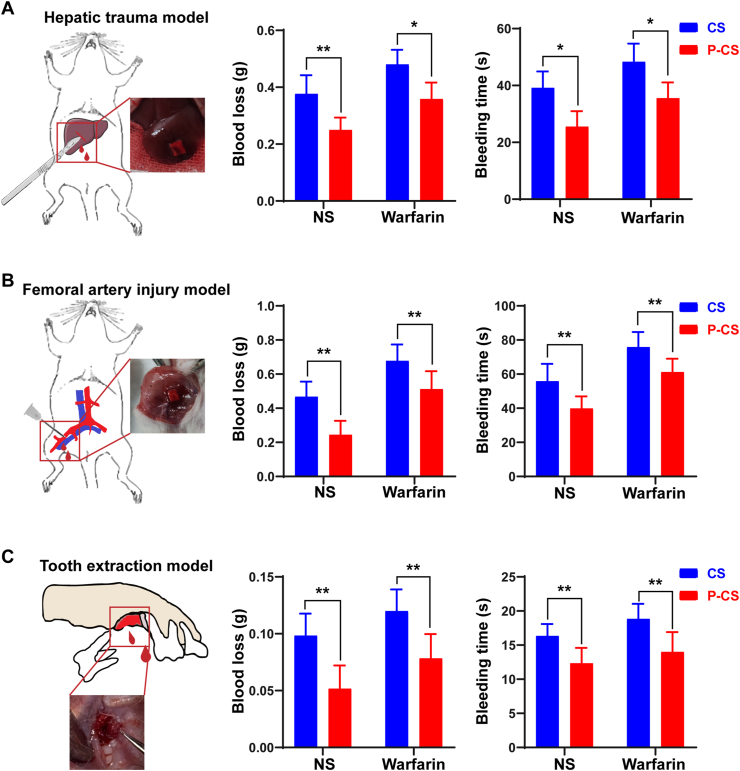
Hemostasis of P-CS *in vivo*. Bleeding time and blood loss of pristine collagen scaffolds (CS) and polyphosphate-crosslinked collagen scaffolds (P-CS) in **A**. the rat hepatic trauma model. **B**. the femoral artery injury model. **C**. the tooth extraction model. Data represent means and standard deviations; ns: no significant difference; *: p < 0.05; **: p < 0.01 (Student's t-test, n = 6).

For the normal saline-treated rats, reduction in blood loss and bleeding time in the P-CS group were statistically significant in all three models (Fig. 4). For the warfarin-treated rats, the P-CS group still showed decreased blood loss and shortened blooding time in the three *in vivo* models (Fig. 4). Thus, P-CS demonstrates favorable hemostatic performance in both healthy rats and rats with coagulation disorder.

### 3.6 *In-situ* bone regeneration

**Fig. 5:**
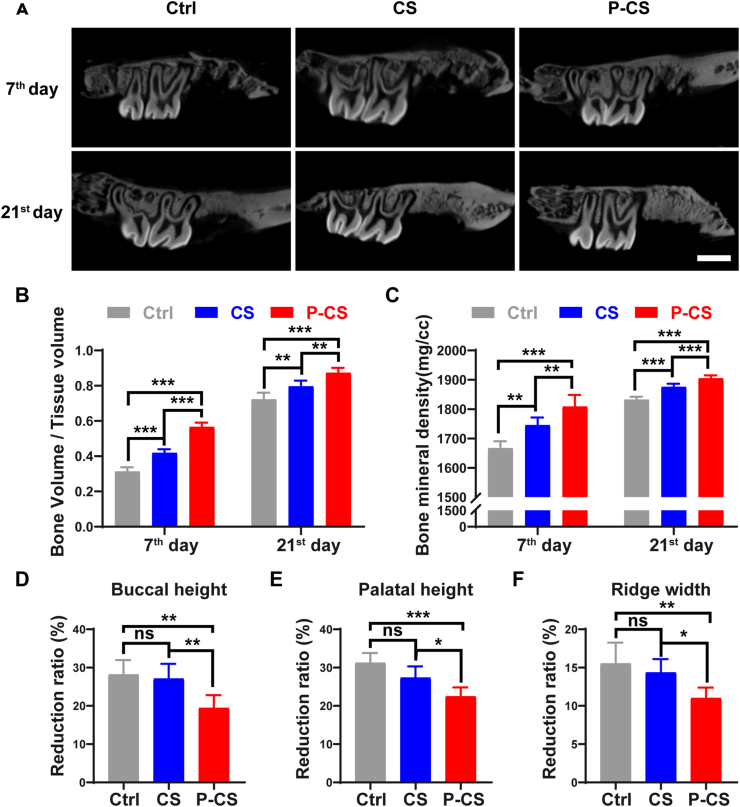
*In-situ* bone regeneration in the rat alveolar bone defect model. **A**. Sagittal view of the rat maxilla on the 7^th^ and 21^st^ day after surgery. Ctrl: control specimens in which defects were not treated with any graft material. CS: pristine collagen scaffolds. P-CS: polyphosphate-crosslinked collagen scaffolds. Bar: 2 mm. **B**. Ratio of bone volume/total volume. **C**. Bone mineral density. For (B) and (C), parameters were calculated based on 3D reconstruction of micro-CT scans. **D**. Buccal ridge height; **E**. palatal ridge height; **F**. alveolar ridge width reduction at 21 days post-surgery. Data represent means and standard deviations; ns: no significant difference; *: p < 0.05; **: p < 0.01; ***: p < 0.001 (one-way ANOVA, n = 6).

Representative micro-CT sagittal sections of maxillary right first molar sockets on 7^th^ day and 21^st^ day after tooth extraction are shown in Fig. 5A. The alveolar bone defect in each group was repaired by newly-formed bone. Both BV/TV and BMD values in the P-CS group were significantly higher at the end of the two time-periods (Fig. 5B and C). Results of statistical analyses of the buccal and palatal ridge height as well as alveolar bone ridge width at the 21^st^ day are shown in Fig. 5D–F. The relevant reduction analyzed in millimeters is shown in Fig. S8. The alveolar ridge height and width of each group were reduced to varying degrees after extraction for 21 days. Nevertheless, the reduction in buccal and palatal height was significantly less in the P-CS group. These results indicate that P-CS reduces morphological changes of the alveolar ridge after tooth extraction.

**Fig. 6:**
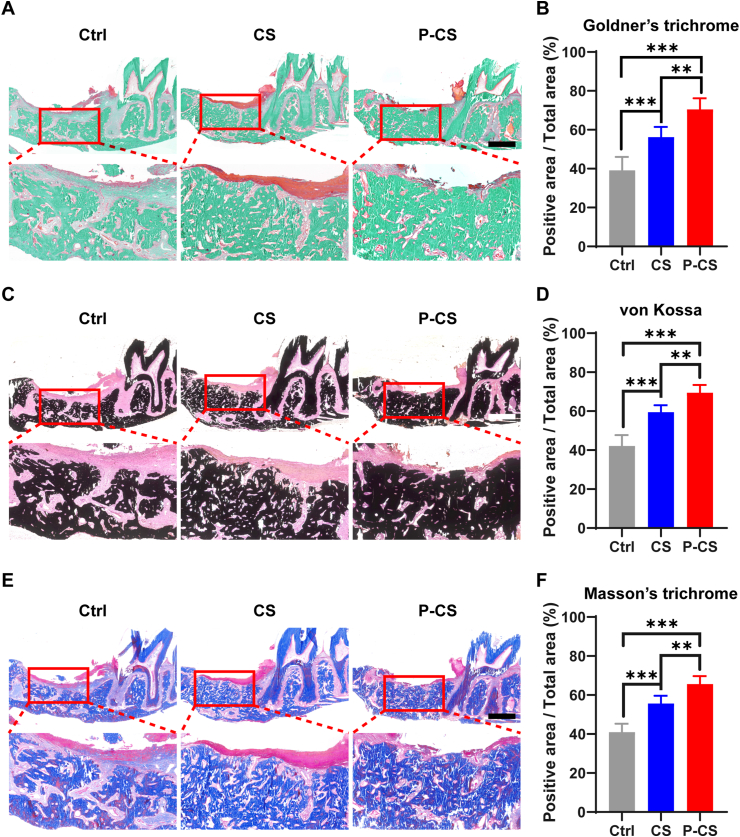
Histological changes in the alveolar bone defect on the 21^st^ day after surgery. Ctrl: control specimens in which defects were not treated with any graft material. CS: pristine collagen scaffolds. P-CS: polyphosphate-crosslinked collagen scaffolds. **A**. Representative images of the bone defect specimens that were stained with Goldner's trichrome. Black bar: 1 mm. **B**. Statistical analysis of the Goldner's trichrome staining results (positive areas/total areas). **C**. Representative images of the bone defect specimens that were stained with von Kossa stain. White bar: 1 mm. **D**. Statistical analysis of the von Kossa staining results. **E**. Representative images of the bone defect specimens that were stained with Masson's trichrome. Black bar: 1 mm. **F**. Statistical analysis of the Masson's trichrome staining results. Data represent means and standard deviations; **: p < 0.01; ***: p < 0.001 (one-way ANOVA, n = 6).

Alveolar bone defect specimens were stained with Goldner's trichrome (Fig. 6A), von Kossa stain (Fig. 6C) and Masson's trichrome (Fig. 6E). There was better formation and mineralization of new bone in the P-CS group. Histomorphometric analysis showed that P-CS group had significantly higher volume of mineralized bone and positively-stained collagen (Fig. 6B, D and F, Table S2). Taken together, these results are indicative of the *in-situ* bone regeneration potential of P-CS.

### 3.7 BMSC behavior on scaffolds with/without blood pre-incubation

**Fig. 7:**
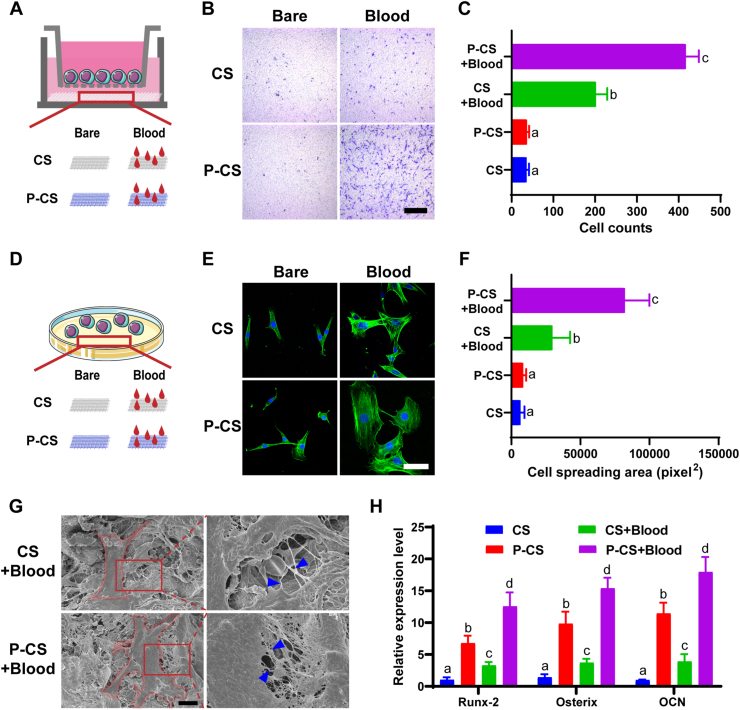
Interaction between blood clots and BMSCs. CS: pristine collagen scaffolds. P-CS: polyphosphate-crosslinked collagen scaffolds. **A**. Schematic of transwell migration assay. **B**. Images (bar: 1 mm) and **C**. quantitative analysis of cell migration in various groups. **D**. Schematic of co-culture of BMSCs with different scaffolds. Cell morphology after incubation for 12 h was detected by **E**. immunofluorescence (bar: 50 μm) and **G**. SEM (bar: 10 μm). The morphology of the BMSCs was outlined in red. The parts of the images outlined by the red squares were magnified. Blue arrows showed contacts of the filopodia with the scaffold matrix. **F**. Cell spreading areas of the BMSCs in the 4 groups were quantitatively analyzed. **H**. Normalized cytoplasmic gene expression of osteogenesis markers after osteogenic induction of the BMSCs for 14 days. For (C), (F) and (H), data represent means and standard deviations. Groups in each chart that labeled with different lowercase letters are significantly different (p < 0.05; two-way ANOVA, n = 6).

Hematoma formation is the first stage of bone healing and the blood clot may influence the behavior of BMSCs. Cell migration was first analyzed by transwell migration assay (Fig. 7A–C). Two-way ANOVA indicated that the cell migration was significantly affected by the factor “blood pre-incubation” (with blood incubation *vs* without blood incubation; p < 0.001) and by the factor “graft material” (pristine collagen scaffold *vs* P-CS; p < 0.001). The interaction of these two factors was statistically significant (p < 0.001). Quantitative results derived from the 4 groups are depicted in Fig. 7C. Within the factor “blood pre-incubation”, significant difference was observed for pairwise comparison between pristine collagen scaffold *vs* P-CS when in the presence of blood pre-incubation (p < 0.001). No significant difference was observed between pristine collagen scaffold *vs* P-CS when without blood incubation (p = 0.9998). Within the factor “graft material”, significant difference was observed for pairwise comparison between with *vs* without blood incubation, irrespective of the graft material [i.e. pristine collagen scaffold (p < 0.001) or P-CS (p < 0.001)]. These results indicate that the blood incubation increased the number of migrated cells, compared with those groups without blood incubation. Moreover, cell migration was not influenced by the graft material (without blood pre-incubation); cell migration was strongly indorsed by the co-effect of P-CS and blood pre-incubation.

Stem cells need to attach to a scaffold after their migration to a bony wound. As shown in Fig. 7D–F, two-way ANOVA indicated that the cell spreading area was significantly affected by the factor “blood pre-incubation” (with blood incubation *vs* without blood incubation; p < 0.001) and the factor “graft material” (pristine collagen scaffold *vs* P-CS; p < 0.001). The interaction of these two factors was statistically significant (p < 0.001). Quantitative results derived from the 4 groups are depicted in Fig. 7F. Within the factor “blood pre-incubation”, significant difference was observed for pairwise comparison between pristine collagen scaffold *vs* P-CS when in the presence of blood pre-incubation (p < 0.001). No significant difference was observed between pristine collagen scaffold *vs* P-CS when without blood incubation (p = 0.9998). Within the factor “graft material”, significant difference was observed for pairwise comparison between with *vs* without blood incubation, irrespective of the graft material [i.e. pristine collagen scaffold (p = 0.0107) or P-CS (p < 0.001)]. These results indicate that the blood incubation increased the spread of cells, compared with those groups without blood incubation. Moreover, cell spread was not influenced by the scaffold material (without blood pre-incubation); cell spread was strongly indorsed by the co-effect of P-CS and blood pre-incubation. Attachment of BMSCs on the two scaffolds that had been pre-incubated with blood was examined with SEM (Fig. 7G). Qualitatively, more contact of the BMSC filopodia with the fibrin network was observed on scaffolds that were pre-incubated with blood.

After osteoinduction for 14 days, BMSCs that were cultured on P-CS exhibited significant upregulations of *RUNX-2, Osterix* and *OCN.* The expressions of those three genes were upregulated after pre-incubation with blood (Fig. 7H). The interaction between these two factors were statistically significant (*RUNX-2,* p = 0.0031; *Osterix*, p = 0.0064; *OCN*, p = 0.0106).

## 4 Discussion

Interventions that can solve both post-extraction bleeding and alveolar bone resorption simultaneously are highly sought after. In the present study, a modified collagen scaffold has been developed by covalently binding polyphosphate onto collagen molecules. The results indicate that P-CS achieves better hemostasis and *in-situ* alveolar bone regeneration without compromising biocompatibility.

The P-CS reduced blood loss and shortened bleeding time in the rat tooth extraction model. This suggests that P-CS is a good treatment modality for arresting post-extraction bleeding. The rat hepatic trauma and femoral artery injury models were used to further confirm the hemostasis potential of P-CS. Similar results were achieved with the tooth extraction model. Because post-extraction bleeding is frequently encountered in subjects with coagulation disorders or those who consume anticoagulants [[2], [3], [4]], a coagulation disorder model was established to examine the hemostatic potential of P-CS in subjects taking anticoagulants. Warfarin is a commonly used oral anticoagulant. The warfarin-treated rats showed prolonged APTT and PT. Hence, the warfarin-treated rats were used in the coagulation disorder model. For the warfarin-treated rats, P-CS also showed reduced bleeding time and blood loss in the three injury models. Taken together, the results indicate that P-CS has excellent hemostasis property in both healthy rats and rats with coagulation disorder.

The enhanced hemostatic potential of P-CS is derived from various aspects. First, the P-CS demonstrated a sharp increase in hydrophilicity, which is an essential factor that contributes to the hemostatic property of a biomaterial. Previous studies improved the hydrophilicity of a material by introducing hydrophilic groups onto the material [33]. The phosphate group is a hydrophilic group [34]. Hence, the increased hydrophilicity of P-CS may be caused by the binding of polyphosphate to the collagen backbone. Increase in hydrophilicity results in better wetting of the surface of the scaffolds, quicker fluid absorption, as well as faster expansion of the volume of a compressed scaffold. These properties help to produce pressure on the injury site and form a concentrated layer of red blood cells to promote hemostasis [21].

The improved hemostasis capability of P-CS may also be attributed to polyphosphate. Polyphosphate consists of linear chains of orthophosphate residues linked by high-energy phosphoanhydride bonds; they are highly-conserved from bacteria to humans [17]. In general, microorganisms contain very long-chain polyphosphates, with sizes ranging from hundreds to thousands of residues. In contrast, eukaryotic cells synthesized much smaller polyphosphates with chain lengths of ∼60–100 units [17]. Compared with the short-chain polyphosphates, the long-chain polyphosphates are potent activators of Hagman factor (factor XII) [35], which initiates the intrinsic coagulation pathway upon surface contact [36]. This is consistent with the result of the present study that P-CS improved hemostasis through the intrinsic pathway. Long-chain polyphosphates are also promoters of platelet activation and thrombin generation [37], which is verified in the present study. The activated platelets release granules containing hemostatic proteins and coagulation factors to further promote clotting [38]. Thrombin is the final effector in coagulation. It activates plasma thromboplastin (factor XI) and augments the intrinsic coagulation cascade [39]. Finally, thrombin converts soluble fibrinogen into fibrin in the common coagulation pathway, producing stable and fracture-resistant blood clots [40]. Hence, the hemostatic mechanism of P-CS may be envisaged as acceleration of the coagulation process via the intrinsic pathway. This is accompanied by the activation of platelet and generation of thrombin.

Apart from promoting hemostasis, P-CS also exhibited favorable alveolar ridge preservation performance. An effective graft material for alveolar ridge preservation requires favorable mechanical strength, resistance to degradation and osteoinductive potential. Collagen has good biocompatibility and osteoinductivity but is poor in mechanical strength and is easy to degrade. Without crosslinking, collagen is unable to maintain the defect space for bone regeneration [14,15,41]. Hence, the present study increased the modulus of elasticity and resistance to degradation of collagen by crosslinking to enable the P-CS to attain the requirement of a biomaterial for alveolar ridge preservation. Although the extraction site achieved primary closure after surgery, the collagen scaffold still has the potential risk of exposure to the oral environment. Both crosslinked and non-crosslinked collagen suffered from poor resistance to degradation when exposed to the oral environment [42]. Investigation of the degradation-resistance of P-CS after exposure to the oral environment is in order.

The enhanced bone regeneration capability of P-CS is also related to polyphosphate. Polyphosphate has been shown to possess favorable bone regeneration activity in various bone defect models [[43], [44], [45]]. It provides phosphate units for calcium phosphate mineralization and delivers chemically-useful energy during enzymatic hydrolysis [17]. It has been reported that polyphosphate induces osteogenesis of BMSCs [19]. This phenomenon was further confirmed in the present study.

The enhanced bone regeneration of P-CS is not only related to polyphosphate, but may also be related to the clots. Bone healing is a complex and well-orchestrated physiological process initiated by the blood clot within a wound [46]. Without a blood clot, bone healing will be significantly delayed [47]. A blood clot functions not only as an initial fibrin scaffold to support cell migration and adhesion, but also as a temporary source of release of growth factors responsible for bone regeneration [48]. As shown in the present study, pre-incubation of collagen scaffolds with whole blood enhanced the migration of BMSCs comparing to the bare collagen. Stem cell migration is possibly influenced by the release of factors from the cellular components of a blood clot [49]. In addition, more cell migration occurred in the P-CS with blood pre-incubation. As shown in Fig. S9, secretion of P-selectin was increased in the blood pre-incubated P-CS group. The promoted cell migration may be related to the enhanced activation of platelets caused by P-CS. The release of growth factors such as insulin-like growth factors and platelet-derived growth factors from activated platelets can stimulate chemotaxis and migration of BMSCs [38]. Identification of the exact growth factors that are responsible for the promoted cell migration is beyond the scope of the present study and requires further investigation.

After migration, the BMSCs need to attach to the fibrin matrix. The fibrin serves as an extracellular matrix for cell adhesion and spreading. In the present study, BMSCs that adhered on blood pre-incubated P-CSs were well-spread, with plenty of filopodia. It has been reported that the fibrin network of a blood clot has thicker fibrils in naturally-healed bone defects, compared with defects with delayed healing [50]. It is envisaged that pharmacological intervention may be used to improve bone healing by forming a thicker fibrin network [47,51]. Thicker fibrin is thought to favor bone healing by aiding cell infiltration and proliferation [52]. The present study showed that thicker fibrin was formed on P-CS because of the presence of polyphosphate [53]. Thus, better spreading of the BMSCs on P-CS may also be attributed to thicker fibrin formation. Because of the increased stem cell migration and adhesion, osteogenesis of BMSCs was also enhanced by the blood clots. The detailed mechanism of improved osteogenesis related to thick fibrin remains to be elucidated.

## 5 Conclusion

Binding of polyphosphate onto a collagen scaffold via “zero-length” crosslinking with EDC/NHS endows the collagen scaffold with enhanced hemostasis and better preservation of the alveolar ridge after tooth extraction. Improved hemostasis is potentially attributed to the ability of polyphosphate to increase the hydrophilicity of the modified collagen scaffold, to activate the intrinsic coagulation pathway and platelets, and to stimulate the production of thrombin. Enhanced alveolar ridge presentation is potentially related to the increase in osteogenesis of BMSCs that promoted by the formation of a high-quality blood clot. The polyphosphate-modified collagen scaffold may be an effective treatment for both post-extraction bleeding and alveolar bone loss. These favorable outcomes should be validated using larger animal models.

## Disclosures

The authors deny any conflict of interest.

## CRediT authorship contribution statement

**Jun-ting Gu:** Conceptualization, Methodology, Investigation, Writing – original draft. **Kai Jiao:** Data curation, Formal analysis, Resources. **Jing Li:** Methodology, Investigation, Software. **Jian-fei Yan:** Validation, Data curation, Formal analysis. **Kai-yan Wang:** Investigation, Validation. **Fu Wang:** Resources, Visualization, Data curation. **Yan Liu:** Resources, Data curation. **Franklin R. Tay:** Writing – review & editing, Supervision. **Ji-hua Chen:** Supervision, Funding acquisition. **Li-na Niu:** Supervision, Funding acquisition, Project administration.

## Declaration of competing interest

The authors declare that they have no known competing financial interests or personal relationships that could have appeared to influence the work reported in this paper.
