# Supplementary material for: Polyphosphate-crosslinked collagen scaffolds for hemostasis and alveolar bone regeneration after tooth extraction
Source: Bioact Mater. 2021 Dec 26;15:68–81. doi: 10.1016/j.bioactmat.2021.12.019 (PMC8940764; doi:10.1016/j.bioactmat.2021.12.019)
Supplement: Multimedia component 1 [file mmc1.docx]

**Supplementary information**

**Polyphosphate-crosslinked collagen scaffolds for hemostasis and alveolar bone regeneration after tooth extraction**

**Fig. S1.** Flow chart depicting the sequence of experiments conducted in the present study.

**Fig. S2.** TEM of ruthenium red stained pristine collagen scaffolds (CS) and polyphosphate-crosslinked collagen scaffolds (P-CS).

**Fig. S3.** XPS spectra of CS and P-CS. The spectra within the black square were magnified.

**Fig. S4.** Biocompatibility of P-CS and CS.

**Fig. S5.** Representative SEM images of blood clots formed on CS and P-CS. Yellow arrow: activated platelets; Bar: 5 μm.

**Fig. S6.** Activated partial thromboplastin time (APTT) and prothrombin time (PT) for warfarin-treated rats (W-rat) and normal saline-treated rats (Ctrl-rat).

**Fig. S7.** Surgical procedure of rat alveolar bone defect model. **A.** Gingiva separation. **B.** Tooth extraction. **C.** Defect creation. **D.** Graft material implantation. **E.** Suture of mucosa.

**Fig. S8.** In-situ bone regeneration in the rat alveolar bone defect model. **A.** Buccal ridge height; **B.** palatal ridge height; **C.** alveolar ridge width reduction at 21 days post-surgery. Data represent means and standard deviations; ns: no significant difference; *: p < 0.05; **: p < 0.01; ***: p < 0.001 (one-way ANOVA, n = 6).

**Fig. S9.** P-selectin secretion in the lower chamber of transwell migration assay. CS: pristine collagen scaffolds. P-CS: polyphosphate-crosslinked collagen scaffolds. Data represent means and standard deviations; ***: p < 0.001 (Student’s t-test, n=6).

**Table S1.** Primer sequences used for RT-PCR.

**Table S2.** Histological changes in the alveolar bone defect on the 21^st^ day after surgery.


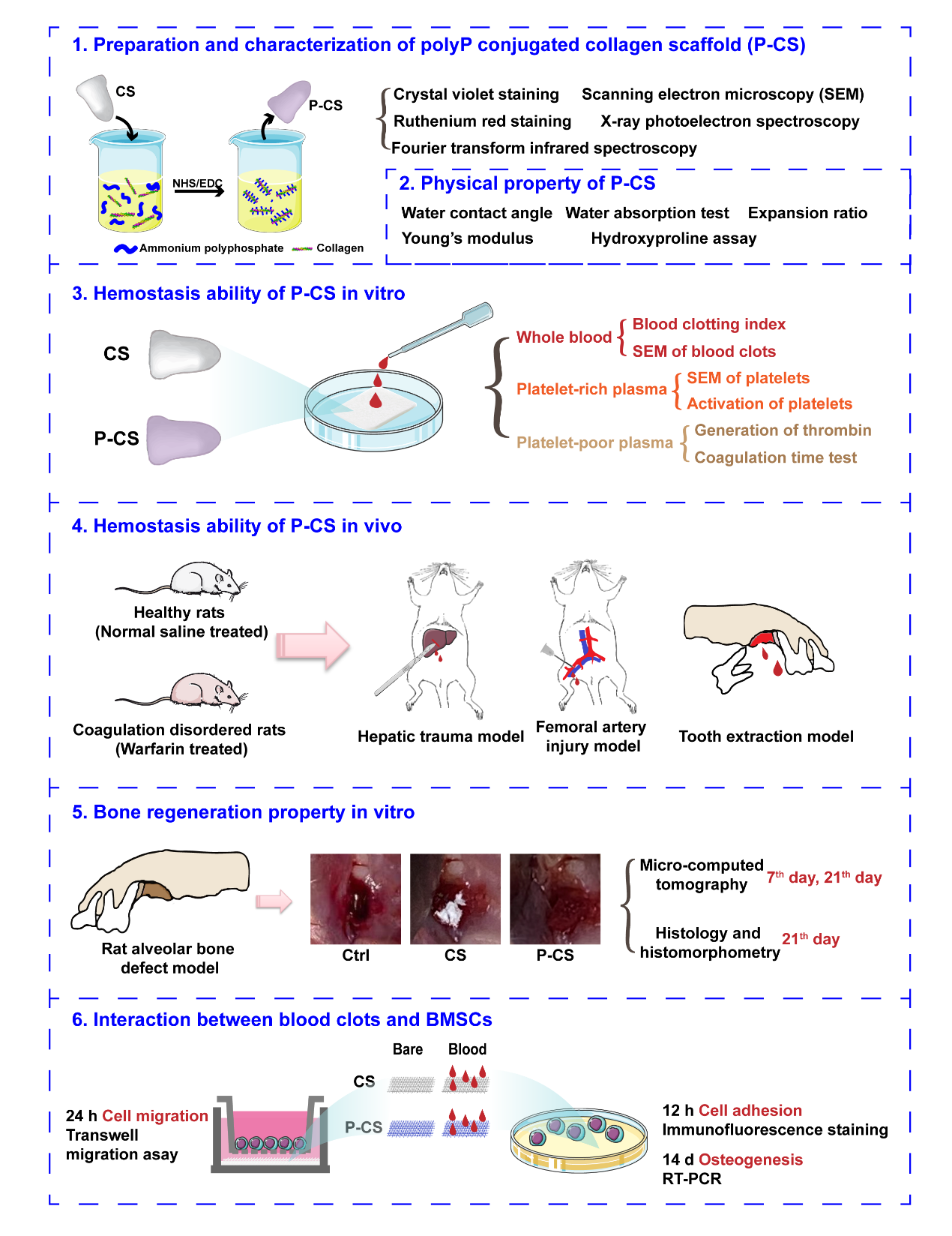


**Fig. S1.** Flow chart depicting the sequence of experiments conducted in the present study.

**Fig. S2.** TEM of ruthenium red stained CS and P-CS. Bar: 400 nm.

**Fig. S3.** XPS spectra of CS and P-CS. The spectra within the black square were magnified.

**Fig. S4.** Biocompatibility of P-CS. **A**. Hemolysis property of CS, P-CS, normal saline solution (NS, negative control) and Triton X-100 (positive control). **B**. Cell viability of different scaffolds. Data represent means and standard deviations. For (A), groups labeled with different lowercase letters are significantly different (p < 0.05; one-way ANOVA, n = 6). For (B), ns represents no significant difference (Student’s t-test, n=6).


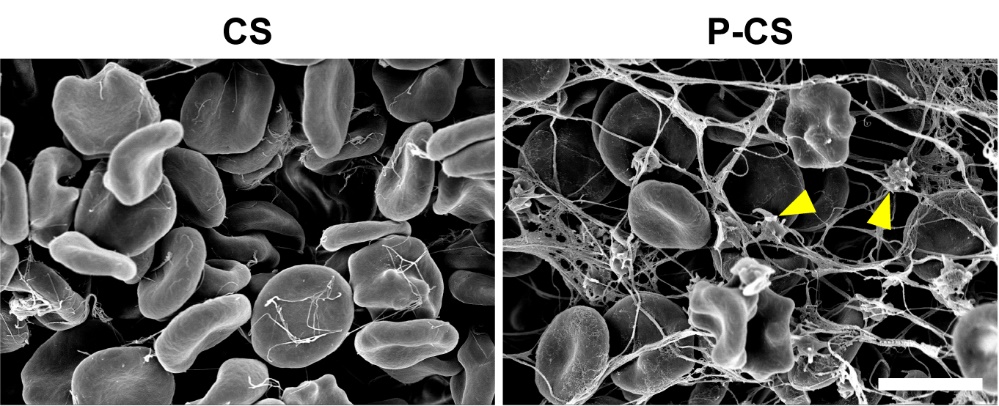


**Fig. S5.** Representative SEM images of blood clots formed on CS and P-CS. Yellow arrow: activated platelets; Bar: 5 μm.


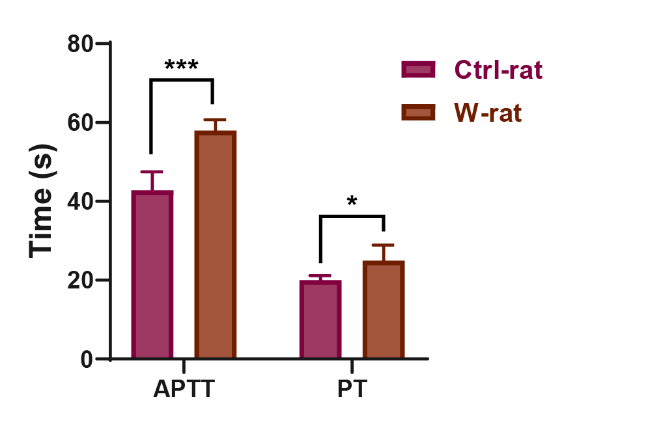


**Figure S6.** Activated partial thromboplastin time (APTT), and prothrombin time (PT) for warfarin-treated rats (W-rat) and normal saline-treated rats (Ctrl-rat). Data represent means and standard deviations; *: p < 0.05; ***: p < 0.001 (Student’s t-test, n=6).


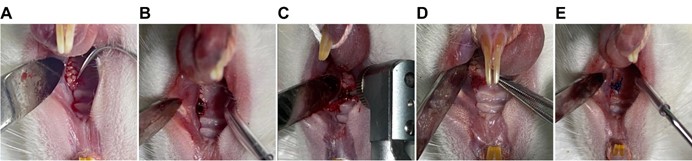


**Figure. S7.** Surgical procedure of rat alveolar bone defect model. **A.** Gingiva separation. **B.** Tooth extraction. **C.** Defect creation. **D.** Graft material implantation. **E.** Suture of mucosa.


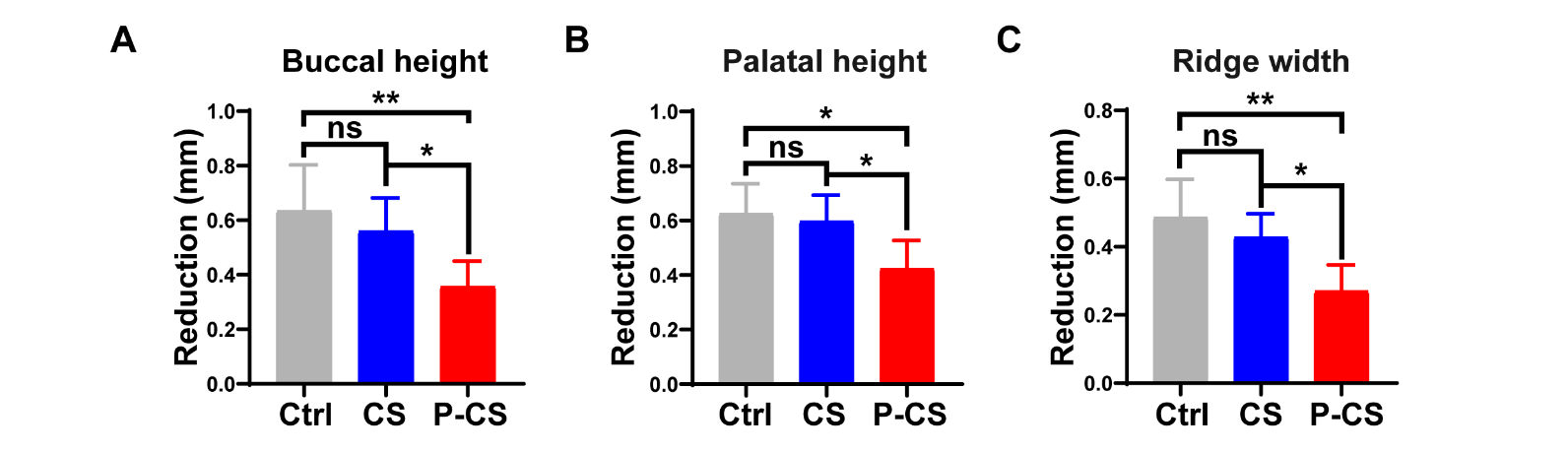


**Figure S8.** *In-situ* bone regeneration in the rat alveolar bone defect model. **A**. Buccal ridge height; **B**. palatal ridge height; **C**. alveolar ridge width reduction in millimeters at 21 days post-surgery. Data represent means and standard deviations; ns: no significant difference; *: p < 0.05; **: p < 0.01 (one-way ANOVA, n = 6).

**Figure S9.** P-selectin secretion in the lower chamber of transwell migration assay. CS: pristine collagen scaffolds. P-CS: polyphosphate-crosslinked collagen scaffolds. Data represent means and standard deviations; ***: p < 0.001 (one-way ANOVA, n=6).

**Table S1.** Primer sequences used for RT-PCR.

| Gene | Primer sequence 5’-3’ |
| --- | --- |
| *Osterix* | \| F:5’-CCTACTTACCCGTCTGACTTTGC-3’ \| \| --- \| \| R:5’-TCCAGTTGCCCACTATTGCC-3’ \| |
| *OCN* | \| F:5’-TGACAAAGCCTTCATGTCCAA-3’ \| \| --- \| \| R:5’-CTCCAAGTCCATTGTTGAGGTAG-3’ \| |
| *Runx-2* | \| F:5’-CCCAACTTCCTGTGCTCCGT-3’ \| \| --- \| \| R:5’-AGTGAAACTCTTGCCTCGTCC-3’ \| |
| *GAPDH* | \| F:5’-CTGGAGAAACCTGCCAAGTATG-3’ \| \| --- \| \| R:5’-GGTGGAAGAATGGGAGTTGCT-3’ \| |

Abbreviations: Forward, F; Reverse, R

**Table S2.** Histological changes in the alveolar bone defect on the 21^st^ day after surgery.

| Group | Mineralized bone by Goldner’s trichrome (%) | Calcified area by von Kossa stain (%) | Collagen by Masson’s trichrome (%) |
| --- | --- | --- | --- |
| Ctrl | 39.14±6.869 | 42.17±5.527 | 40.93±4.344 |
| CS | 56.20±5.258 | 59.44±3.619 | 55.61±3.983 |
| P-CS | 70.49±5.700 | 69.48±3.949 | 65.62±4.062 |

Ctrl: control specimens in which defects were not treated with any graft material. CS: pristine collagen scaffolds. P-CS: polyphosphate-crosslinked collagen scaffolds.
